# The Year in Cognitive Neuroscience

**DOI:** 10.1111/nyas.12110

**Published:** 2013-04-30

**Authors:** Sven Bestmann, Eva Feredoes

**Affiliations:** 1 Sobell Department of Motor Neuroscience and Movement Disorders, UCL Institute of Neurology, University College LondonLondon, United Kingdom; 2 School of Psychology and Clinical Language Sciences, University of ReadingReading, United Kingdom

**Keywords:** state-dependence, effective connectivity, transcranial magnetic stimulation, causal inference, EEG, fMRI, MRS, computational neurostimulation

## Abstract

Modern neurostimulation approaches in humans provide controlled inputs into the operations of cortical regions, with highly specific behavioral consequences. This enables causal structure–function inferences, and in combination with neuroimaging, has provided novel insights into the basic mechanisms of action of neurostimulation on distributed networks. For example, more recent work has established the capacity of transcranial magnetic stimulation (TMS) to probe causal interregional influences, and their interaction with cognitive state changes. Combinations of neurostimulation and neuroimaging now face the challenge of integrating the known physiological effects of neurostimulation with theoretical and biological models of cognition, for example, when theoretical stalemates between opposing cognitive theories need to be resolved. This will be driven by novel developments, including biologically informed computational network analyses for predicting the impact of neurostimulation on brain networks, as well as novel neuroimaging and neurostimulation techniques. Such future developments may offer an expanded set of tools with which to investigate structure–function relationships, and to formulate and reconceptualize testable hypotheses about complex neural network interactions and their causal roles in cognition.

## Introduction

Over the past two decades, neurostimulation approaches have become successful tools for noninvasively studying the basic physiology of, and cognitive processes in, the human brain. This review discusses how the use of neurostimulation in combination with neuroimaging has answered questions about the relationship between the physiological impact of transcranial magnetic stimulation (TMS) and its behavioral consequences, the distributed impact TMS can have on functional brain networks, and how this impact can be exploited to address novel questions about causal network interactions underlying cognition.

The format of this paper prevents an exhaustive treatment of combinations of neurostimulation and neuroimaging, and therefore the focus here is on TMS and its concurrent combinations with functional magnetic resonance imaging (fMRI) and electroencephalography (EEG). Other neurostimulation techniques provide equally powerful means for transiently interacting with neural processing. Of these, transcranial direct current stimulation (tDCS) stands out in its capacity to interact with ongoing neuronal activity.^1^–^3^ Although the relative paucity of combined tDCS and neuroimaging studies prevents an in-depth review of the technique,^4^–^8^ many of the arguments presented here equally apply to tDCS. We furthermore focus on concurrent combinations, and for elegant offline work, recent reviews and examples are referred to.^9^–^19^ Focusing on fMRI and EEG, the examples discussed in this review establish principles and ideas that similarly apply to other imaging modalities. Finally, for safety considerations,^14^,^20^,^21^ technical and methodological details, and requirements for combining TMS with neuroimaging, the reader may refer to previous publications.^14^,^22^–^36^

The below discussion is divided into three parts, the past, present, and potential future contributions to the field. First, early work, particularly in the motor system, is reviewed that provided evidence for the anatomically distributed effects of TMS. This in turn led to the combination with neuroimaging to study such effects with high anatomical and temporal precision throughout the brain. Since its implementation, the combination of TMS with neuroimaging has established several, now widely accepted insights into the basic mechanism of action of TMS and its distributed impact on brain networks. Next reviewed are more recent examples of combined TMS–fMRI and TMS–EEG that demonstrate how interregional interactions depend not only on anatomical connectivity, but also on the activation state of network constituents. A key question when evaluating successful combinations of TMS and neuroimaging concerns their ability to formulate novel and testable hypotheses about the role of distributed brain networks for cognition. As argued in the final section, the field is at a segue: the potential of multimodal neurostimulation approaches for understanding human cognition has not been fully exploited, but recent developments provide an ever increasing arsenal of possibilities for their use in establishing network accounts of human cognition.

## The past: resting state studies and perturb-and-measure approaches

TMS stimulates cortical tissue through electromagnetic induction by discharging a short (∼1 ms) but strong (several kA) electrical current through an induction coil, which is placed over a cortical region of interest. The electric pulse induces a time-varying magnetic field perpendicular to the stimulation coil that passes through the scalp without attenuation, and is therefore painless and well tolerated. The induced electrical current may directly interact with ongoing neural processing at the site of stimulation, but also in remote and connected brain regions, as discussed in further detail below. By having such direct input into a cortical operation, one can study its behavioral consequences and thereby ask questions about the requirement of ongoing activity for a cognitive operation. Early seminal studies by Amassian *et al*.^37^,^38^ in the visual system provided the first compelling examples of how the possibility to directly and noninvasively interact with cortical processing can be hedged to infer causal structure–function relationships. In brief, these studies demonstrated, with a high degree of both temporal and anatomical specificity, that stimulation of early visual cortex can transiently interfere with perception.^37^,^39^,^40^ Early studies on human motor cortex, on the other hand, have provided the first evidence that TMS is capable of impacting distal sites. For example, a single pulse applied to the primary motor cortex (M1) hand representation can elicit motor-evoked potentials (MEPs) in muscles of the contralateral hand.^41^–^46^ The generation of MEPs involves (at least) three stages where signals are relayed first via synapses onto corticospinal neurons, then via synapses onto motor neurons located in the spinal cord, and finally, by the neuromuscular synapses that generate the evoked potentials recorded from peripheral muscles.^47^ TMS to M1 also significantly influences activity in the contralateral homologue,^48^–^50^ with functional relevance to motor output.^51^

Behaviorally, the first double-coil TMS studies provided a similar picture. For example, stimulation of visual area V5/MT with one TMS coil was reported to influence the excitability of primary visual cortex, as assessed with subsequent TMS pulses applied with a second stimulation coil.^52^ Functionally, this remote influence significantly affected visual awareness,^52^ thus providing additional evidence that even single TMS pulses can elicit remote responses strong enough to shape behavior. Although the relationship between the physiological and behavioral consequences resulting from TMS are more complex than simply that of transient interference,^53^–^55^ it is clear that TMS has the capacity to provide focal and temporally precise inputs into the operation of a cortical region,^53^ with highly specific behavioral consequences.^56^

**Figure 1:**
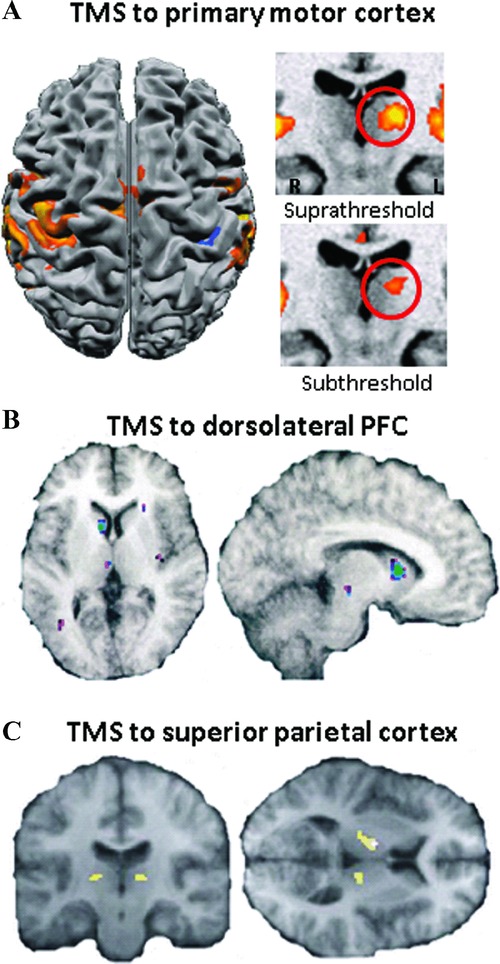
Subcortical activity changes evoked by TMS as measured using fMRI and PET. (A) Brief bursts of rTMS to the left primary motor cortex (M1) evoke BOLD signal changes not only in the vicinity of the stimulation site but additionally in the ipsilateral motor thalamic nuclei (note: left and right reversal). These subcortical effects occur even during subthreshold TMS and therefore in the absence of evoked movements.^68^ (B) Changes in extracellular dopamine concentration measured *in vivo* using [(11)C]raclopride and positron emission tomography. Repetitive TMS of the left dorsolateral prefrontal cortex caused a reduction in [(11)C]raclopride binding in the left dorsal caudate nucleus compared with rTMS of the left occipital cortex.^78^ (C) Interaction of TMS (high and low intensity) and median nerve stimulation (ON and OFF) within the thalamus. BOLD signal in the thalamus was highest during combined right-hand somatosensory stimulation and high-intensity TMS over the right parietal cortex.^110^

But real progress in our understanding of local and remote TMS effects came with the near-simultaneous advent of combinations of TMS combined with fMRI,^57^ EEG,^58^ and positron emission tomography (PET).^59^ Early studies employed perturb-and-measure approaches^60^ in which TMS is used at rest to cause activity in one brain region while concurrent neuroimaging characterizes the distributed impact of this intervention. Two findings of these early resting-state TMS–neuroimaging studies are emphasized here, as they have particular relevance for later studies on cognition. First, the early combinations, using both single pulses or short bursts of TMS to motor cortical regions, established the now accepted view that TMS can interact with distal sites, including subcortical structures (Fig. 1),^61^–^68^ in a dose-dependent manner.^61^,^63^,^68^–^74^ Such interactions might be predicted given the strong anatomical projections across different corticobasal ganglia–thalamic loops,^75^–^77^ but direct quantification of such interactions had not been possible until the combination with neuroimaging. Even more specific cortico–subcortical interactions were demonstrated with combinations of TMS and ligand-PET (albeit conducted with offline TMS). These showed, for example, that stimulation of the dorsolateral prefrontal cortex (DLPFC) can elicit changes in dopamine release in the caudate nucleus (Fig. 1),^78^ or in the putamen after M1 stimulation.^79^ Similarly, concurrent TMS–fMRI studies showed that changes in subcortical activity during M1 stimulation can be distinguished from those evoked by stimulation of the dorsal premotor cortex (PMd), despite considerable and expected overlap of their anatomical footprints.^61^,^68^

Second, resting-state TMS–EEG studies have provided clear demonstrations that the impact of TMS on remote regions is rapid and can spread over relatively widespread areas of cortex even with administration of single pulses or short stimulation bursts.^71^,^80^–^87^ Indeed, TMS-evoked activity spreads within tens of milliseconds to directly adjacent cortical regions, but then quickly disperses to more distal sites in a reverberating pattern.^58^,^82^,^88^ The magnitude of this spread is dose dependent,^71^,^89^ and can also be observed even after single pulses of stimulation.^58^,^90^ The changes in network activity observed with neuroimaging in response to single pulses or short bursts of TMS are thus not compensatory or plastic adjustments (as those likely induced by offline repetitive TMS protocols), but instead reflect the immediate propagation or broadcasting of the induced activity to distal, but connected, sites.

Together, this early combined resting-state neuroimaging–TMS work provided empirical confirmation that the distributed impact of TMS is spatially and temporally specific and occurs within distinct anatomical networks.^a^ It is important to remember that this approach departs from the use of TMS in many behavioral applications, where it is aimed at transiently and reversibly disrupting behavior to induce virtual lesions. Instead, TMS is now used to cause activity in a cortical region, and measure with neuroimaging how this change is broadcast to distal regions, including subcortical sites. Without such combinations, our inferences would largely be confined to the cortex directly underneath the stimulating coil (although we note that double-coil TMS approaches also provide powerful ways for looking at interregional influences on, for example, M1 or V1^52^,^91^–^97^).

Two key issues arise: first, earlier electrophysiological and behavioral work has shown that the impact of TMS depends on the excitability of connections (and/or the current level of activity) at the time of stimulation. Put simply, the more excitable a given region or its connections are, the more likely it is that TMS will influence activity in local and distant brain regions. For example, applying TMS to M1 during voluntary contraction affects the size and number of descending corticospinal volleys.^98^–^100^ This state-dependence of TMS is also supported by studies in the visual cortex demonstrating that the intensity of TMS-evoked perceived light flashes (phosphenes) changes during migraine,^101^ spatial attention,^102^ or neural adaptation paradigms.^103^–^105^ The next section will address how combined TMS and neuroimaging has helped to understand state-dependent network interactions.

The second issue is to what degree combined TMS and neuroimaging has not just enriched our understanding about the mechanisms of action of TMS itself, but has also provided novel views on the role of network interactions in human cognition, which is addressed in the final section.

## The present: state-dependent network effects and oscillatory changes

Recently published studies combining TMS with neuroimaging extend the earlier work described, now asking about changes in the influence of TMS-targeted brain regions on anatomically remote regions that also change as a function of the behavioral requirements. The spread of TMS-induced activity to functionally connected areas is therefore a means for probing the varying states of connectivity, as stimulation can be applied during different behavioral states, at different times, to different areas. This approach is briefly illustrated using examples in which TMS was combined online with fMRI or EEG. We particularly acknowledge here the significant contribution Jon Driver made to the combined TMS–neuroimaging field; he was very much at the forefront of applying this approach to investigate a variety of cognitive domains, and his success in doing so is made apparent in this section.

### State-dependent interhemispheric interactions

**Figure 2:**
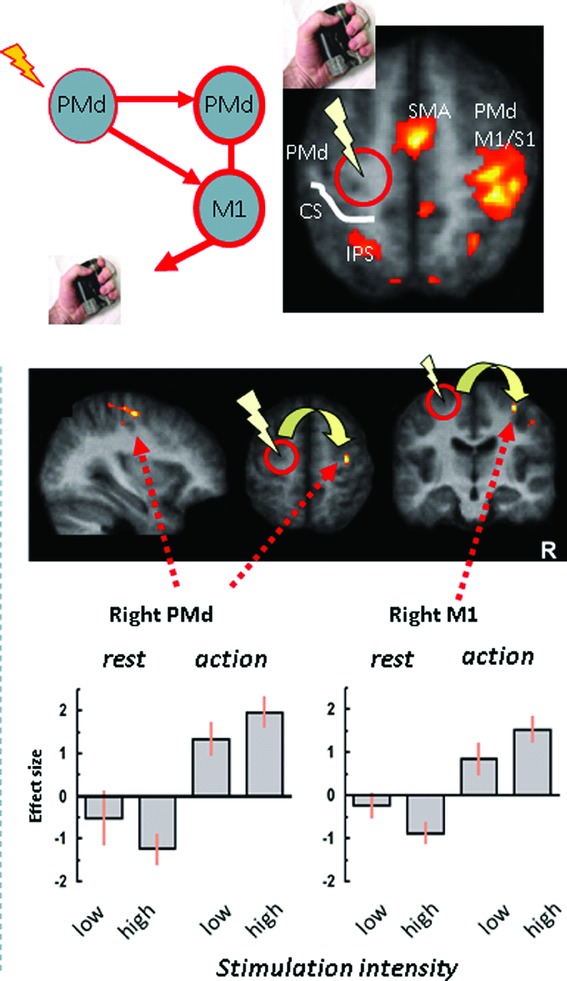
State-dependence of interhemispheric influences of TMS in the motor system. Short bursts of TMS (high vs. low intensity) were applied to the left PMd during left-hand isometric force production (or nonmotor rest), concurrently with fMRI. Within the task-related right PMd and M1 (i.e., contralateral to TMS, top), TMS at high intensity leads to a relative activity increase (bottom), compared to low-intensity control TMS. However, this effect is reversed during nonmotor rest, with high TMS now leading to a relative activity decrease in contralateral motor regions.^108^

One striking result from early resting-state TMS–neuroimaging combinations was the rapid and reliable spread of effects to the hemisphere contralateral to the stimulation site.^58^,^61^–^63^,^68^,^106^ The nature of such interhemispheric interactions among homologous premotor and M1 areas was the focus of a concurrent TMS–fMRI investigation of simple force production.^107^ Specifically, this study addressed the hypothesis that the PMd might increase its influence with the contralateral PMd and M1 when a voluntary motor action is performed, as opposed to when at rest. By applying a short burst of TMS to the left PMd, its influences on contralateral motor regions during isometric force production or rest were measured. TMS produced a differential effect on blood oxygenation level–dependent (BOLD) signal changes both in the stimulated area, and in the contralateral right PMd and M1. Critically, these effects depended on the behavioral state (voluntary grip force production versus rest); at rest, TMS led to a relative BOLD signal decrease in these regions, whereas during grip, a relative increase was observed (Fig. 2). The remote influences of TMS on a known motor network therefore depend on the trial-by-trial changes in the state of the targeted region at the time of stimulation, and can even reverse with changes in state. This finding suggests that specific brain regions within a functional network only influence one another when a specific behavioral context is present, shown here as influences among contralateral and homologous brain regions.

This work was extended using TMS in combination with arterial spin labeling,^108^ an MRI approach that measures cerebral blood flow,^28^ now asking about the role of the PMd in the mapping of prelearned associations to motor responses. TMS produced regional cerebral blood flow changes (rCBF) on a task requiring sequences of previously learned key presses in response to visual stimuli. Compared to free selection of key presses, TMS increased rCBF in the contralateral PMd and other regions of the motor network, indicating a role for the PMd in the mapping of external cues to motor movements, via the formation of a transient functional network.

Finally, in another concurrent TMS–fMRI study, TMS applied to the right parietal cortex during wrist nerve stimulation (compared to no stimulation) produced increased activity in the contralateral primary somatosensory cortex and subcortically in the thalamus. This provides another example for causal interhemispheric interactions, now in the somatosensory system.^109^ The state-dependent effects support a role for the stimulated parietal region in response enhancement via a corticothalamic circuit when somatosensory inputs are present. Collectively, these examples all show the utility of combining online TMS with fMRI to address specific questions related to the interplay between regions in a functional network that can involve brain regions contralateral to the point of stimulation.

### Anatomically distributed state-dependent and top-down influences

More recent work has started to use concurrent TMS–fMRI to investigate state-dependent interregional interactions outside the motor system. Note, however, that recent examples in the visual domain are discussed here only in passing; for an in-depth discussion of top-down visual influences, readers can refer to the contribution by Ruff (this volume).

To investigate top-down influences in the domain of multisensory integration, TMS has been applied to an association area (intraparietal sulcus; IPS) to examine TMS-modulated feedback to primary sensory cortex during auditory, visual, or no external stimulation.^110^ Participants were presented with different sensory stimulation during which effective, ineffective, or no TMS was applied to the right IPS. State-dependent effects resulting from visual stimulation were produced in primary sensory areas; IPS-TMS increased BOLD response in visual areas, again supporting a role for this area in response amplification/enhancement. When auditory or no external stimulation was present, IPS-TMS instead revealed cross-modal effects, in which early visual cortex activity decreased, in addition to expected increases in auditory cortex activations. These results suggested a role for the IPS in sensory gain control or modulation of interactions between different sensory cortices.

The role of the parietal cortex in attention has also been studied with concurrent TMS–fMRI.^111^,^112^ In these investigations, the top-down influence of parietal sites over visual cortex was shown, in which TMS applied during the directing of covert attention toward one hemifield increased BOLD signal in early visual areas. These findings show the causal influence of parietal over visual areas during varying states of attention, and fit with models in which modulatory effects of spatial attention on visual cortex occur via effective connectivity with parietal regions. More detailed descriptions of these studies, and more examples of using TMS–fMRI to study top-down effects in the visual system, are provided by Ruff (this volume).

**Figure 3:**
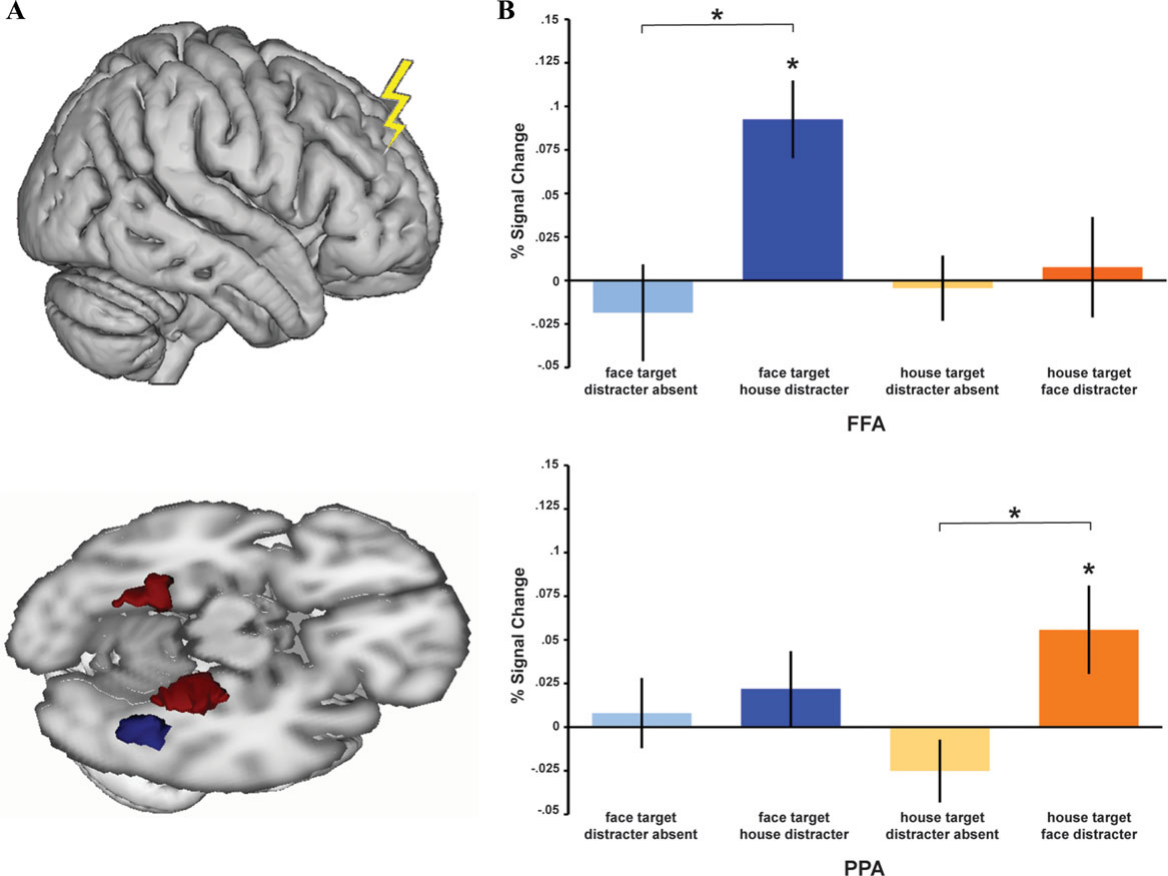
Anatomically remote effects of TMS to reveal the mechanisms of DLPFC-based control on WM representations. (A) Schematic of the right DLPFC stimulation site (upper), a region involved in distractor mitigation during WM. The impact of stimulation was assessed in posterior visual category-specific areas (lower; fusiform face area (FFA) in red, parahippocampal place area (PPA) in blue)). (B) Interparticipant mean BOLD percent signal changes due to effective vs. ineffective DLPFC-TMS are shown in FFA (upper) and PPA (lower). Effective TMS increased BOLD in FFA specifically when faces were memory targets, in the presence of house distractors. Analogously, effective TMS increased activity in PPA when houses were memory targets and faces were distractors. Thus, DLPFC stimulation has an impact on the posterior region representing the current memory target (rather than the current distractor), but only in the presence of distraction.^114^

In another investigation of how a top-down control region exerts control over anatomically remote visual areas, concurrent TMS–fMRI was used to resolve the question of whether the DLPFC mitigates distraction during working memory (WM), through an enhancement of relevant memory targets or suppression of irrelevant distractors.^113^ To this end, during fMRI scanning, TMS was applied to the right DLPFC to coincide with distractors, and the resultant effects of TMS on the BOLD response were measured in remote visual areas responding to either memory targets or distractors; in this way, the recipient of the DLPFC control signals (memory targets or distractors) would be revealed. TMS increased BOLD signal in memory target regions only, providing support for an enhancement mechanism of top-down DLPFC-mediated control (Fig. 3). This result fits with previous findings in which relevant (over irrelevant) information for the task is preferentially targeted by DLPFC signals.^114^ Moreover, there was no effect of TMS on memory target regions in the absence of distractors, providing further constraints on the role of the DLPFC during WM (i.e., only when the contents of WM need protection from external distraction). In sum, these results provide a strong line of causal evidence on the conditions under which the DLPFC is effectively connected to visual regions representing memory targets during WM, with additional conclusions able to be made on the mechanism by which a control region protects memory targets (i.e., via their enhancement). More generally, this study is an elegant example of the inferential power provided by concurrent TMS–fMRI, which has moved beyond simply assessing which regions are functionally coupled during a task. The nature of the TMS effect (i.e., an increase in BOLD signal in target-relevant regions) suggests a mechanistic action of DLPFC-based control, providing a unique form of evidence that is consistent with those observed in single unit recordings in monkeys, previous fMRI studies,^115^ and models of (prefrontal-based) cognitive control.^116^,^117^

Of note for the majority of the studies described above is the use of TMS during fMRI in physiological probe mode (i.e., without interfering with behavior). The reasons for this are often practical; for most behaviors, more TMS than permitted by the constrained TMS–fMRI environment must be given to significantly interfere with behavior; but by not disturbing behavior, networks can be investigated under comparable behavioral conditions. At the same time, the absence of behavioral consequences of TMS may instead, or at least under certain conditions, be due to rapid compensatory changes that counteract the stimulation. This now opens the possibility to ask about the specific brain regions that may enable such rapid compensatory adjustments, and at which point in the information processing stage such adjustments may occur.^118^ Furthermore, by not modulating behavior, one of the strengths of TMS is lost: the causal conclusions that can be drawn when a disrupted brain region impairs performance of the cognitive operation of interest. In a rare example of TMS interference during concurrent fMRI^119^ (see also Ref. 112 for a similar approach), right (but not left) parietal TMS during visuospatial task performance increased reaction times, with concomitant decreases in BOLD signal in the stimulated region and also in the right medial frontal gyrus. The task-specific (i.e., state-dependent) effect of parietal TMS on the frontal cortex raised the question of whether this disruption contributed to the behavioral impairments observed. One indication that this may have been the case was that TMS-evoked activity changes in the right frontal and parietal cortex correlated with the behavioral impairments.

Another approach for disrupting behavior that circumvents fMRI-imposed constraints is to use an offline TMS protocol, applied immediately before scanning. Such offline approaches to investigate compensatory or adaptive processes are not discussed here, but for recent examples using this approach to investigate the connectivity and mechanistic actions of the prefrontal cortex on posterior visual perception–related regions, see Refs. 12 and 120.

### TMS-induced oscillatory changes

TMS pulses can be applied rhythmically at frequencies to match those of endogenous oscillations, and this capacity offers the potential to causally unlock the cognitive role of oscillations. Of particular interest has been the role of α band oscillations. Traditionally known as the idling rhythm, α has been associated more with a resting-type state than with active cognitive operations.^121^ This view has undergone a recent shift, of which evidence using combined TMS–EEG has contributed. For example, a recent study demonstrated a dependence of the preceding α phase on the efficacy of phosphene production by a single TMS pulse applied over the occipital cortex.^122^ For frontal and occipital electrodes, the phase of oscillations in the α range was systematically coupled with the probability of phosphene report by participants for a period of up to 400 ms prior to phosphene induction, a result that provides causal evidence for ongoing α oscillations in sensory perception (see also Ref. 123). This idea has been further consolidated in recent combined TMS–EEG work on α oscillations and perception, now applying TMS to the IPS during a Posner cueing task to investigate the interaction between α and endogenous allocation of attention.^124^ Specifically, both the left and right IPS were targeted during the presentation of a cue indicating the location of an upcoming visual target. TMS interfered with subsequent target detection and also disrupted occipito-parietal α desynchronization in the hemisphere contralateral to the cue, supporting a role for this region in the allocation of spatial attention to one hemifield. However, right IPS TMS had an additional effect of synchronizing α rhythms bilaterally, suggesting a spatially nonselective role for α as well, possibly to control visual cortex excitability prior to an upcoming target, a result that is also in agreement with parietal asymmetries revealed by concurrent TMS–fMRI.^73^,^125^

**Figure 4:**
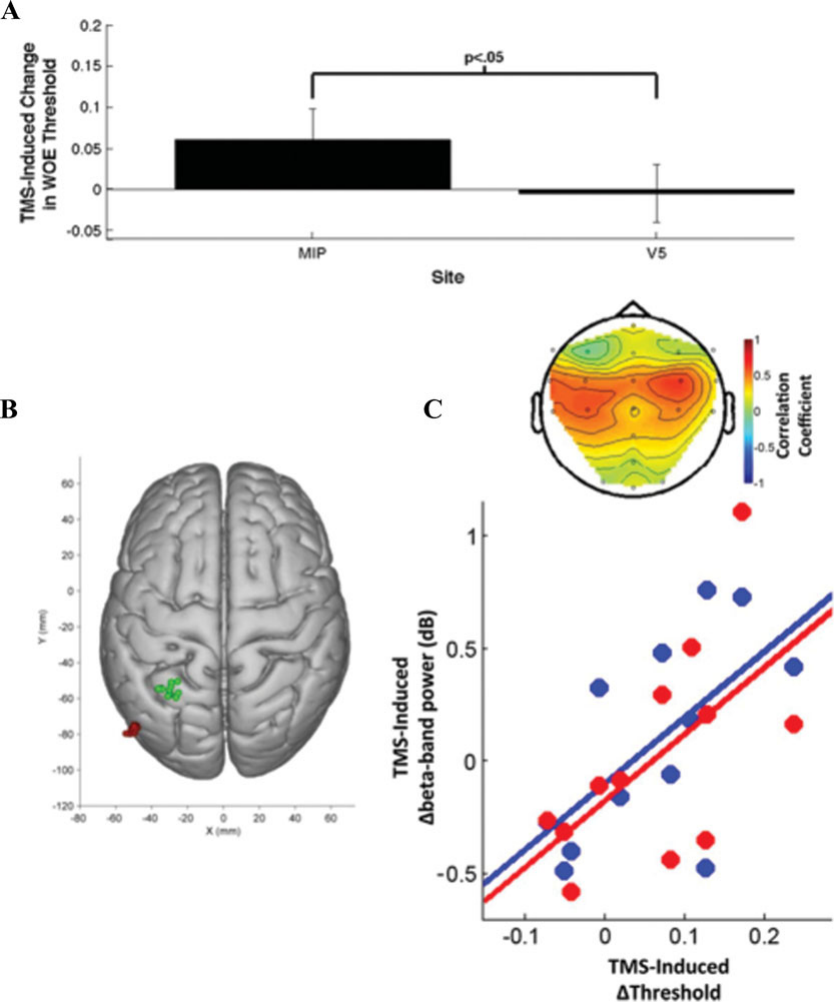
Effects of intraparietal TMS during perceptual decision making on sensorimotor oscillatory activity. (A) TMS-induced change in psychometric function during a perceptual decision-making task, for the left medial intraparietal area (MIP; green) vs. V5 (red) control stimulation (B) revealed a specific correlation between TMS-induced behavioral changes during a perceptual decision task, and concurrently measured β-band changes for electrode positions overlying sensorimotor cortex in both hemispheres (C).^127^

It has been suggested, however, that α oscillations might not just be related to processing in the visual domain, but may also play an important role in decision making. Recent work has elegantly addressed the question regarding specific roles for α and β oscillations in a probabilistic reasoning task.^126^ Using EEG, a recent study demonstrated that the arrival and accumulation of evidence about an upcoming decision, which was indicated via left or right hand button press, was indexed by α and β oscillations in sensorimotor cortex.^126^ To causally establish the role of these oscillatory signatures for evidence accumulation and decision making, a subsequent experiment applied short trains of 10 Hz TMS, during EEG, over a region of the left IPS where sensorimotor implementation of the decision was expected to occur (Fig. 4). TMS now biased decision responses toward the left hand, indicating disruption of evidence integration for responses that were to be given from the contralateral-to-TMS hand. TMS also affected β band power, which, after TMS, showed a positive correlation with an individual's decision threshold bias. Moreover, the largest β power increases occurred when TMS was applied at a specific phase of the β cycle. No effects of TMS were observed on α, however. These results thus provide a novel form of evidence for the causal role of β oscillations in the processes underlying an upcoming decision, perhaps reflecting the incoming signals tracking probabilistic information that goes toward response selection, with the IPS playing a critical role.

Turning once again to WM, a role for oscillations in WM storage has also been proposed,^127^–^129^ which was directly tested using combined TMS–EEG.^130^ During the maintenance period of a spatial or object WM task, α frequency (10 Hz) repetitive TMS was applied over the superior parietal lobule (SPL), which was previously shown to be affected by TMS during spatial WM.^131^ Correlations between TMS-induced changes in task performance and α band power were identified in several (source-localized) brain areas related to spatial WM, with TMS-induced impairment in spatial WM accuracy corresponding specifically to decreases in α band power. Cross-frequency effects between α and γ were also modulated by TMS, adding causal support for a proposed relationship between these frequency bands.^132^ In addition to demonstrating specificity for certain frequencies in WM, these results also demonstrated the biasing of endogenous oscillations by rhythmic TMS. Another study also used combined TMS–EEG to help clarify the interaction between endogenous behavioral states of WM and exogenously induced electrophysiological effects by TMS, producing effects on a range of EEG measures, including event-related potentials and oscillations, that were consistent with both behavioral state and the underlying physiological state of the cortical target at the time of stimulation.^133^

Several studies have used TMS to interact more explicitly with endogenous oscillations related to cognition to show the ability of TMS to bias, or entrain, specific frequencies. An excellent example is provided by a study^134^ that used concurrent TMS–EEG to entrain and measure a cortical generator of α oscillations related to shifts of attention and target detection. TMS pulse trains were applied at a frequency corresponding to each subject's individual α, with resultant effects on endogenous α from each pulse, measured concurrently with EEG. Against a series of control conditions, their results showed that α-frequency trains of TMS started off with broadband, and topographically broad, increases in oscillatory power that narrowed to the α range as the TMS train progressed in time. The phase of an individual's intrinsic α cycle immediately prior to the start of a TMS train also influenced TMS-induced entrainment, with the strongest entrainment observed when TMS pulses were phase locked to a specific period in the α cycle. This phase locking increased as, again, the number of TMS pulses applied in a train increased. These results complement earlier work,^127^,^129^ and provide yet more evidence of the causal effect that rhythmic TMS has on intrinsic oscillations; TMS can synchronize oscillations and, as shown previously in a visual detection task, this entrainment can have behavioral consequences.^135^ More recent developments in this area are discussed in the following section.

## The future: can neurostimulation be an arbitrator for network accounts of cognition and their physiological underpinnings?

Next considered is how the field of cognition with neurostimulation perhaps can and should progress. Combined neurostimulation and neuroimaging applications for studies of cognition must be judged by their ability to refine or reshape cognitive models by exploring their implementation via neuroanatomical and neurophysiological underpinnings. Despite the success of multimodal neurostimulation approaches, we argue that the impact of combined neurostimulation and neuroimaging has yet to reach its full potential when it comes to informing neural models of cognition.

### TMS as arbitrator for neural models of cognition

Perhaps owing to the complexity of the TMS-evoked physiology and the methodological prowess required to conduct combined studies, much of the work to date has been geared toward the understanding and characterization of the basic physiology and state-dependence of TMS effects in distributed networks, all prerequisites for an in-depth understanding of the impact of TMS (and indeed all forms of neurostimulation) on cognition. One highly constructive role for TMS–neuroimaging in particular may be that of an arbitrator, in which the interventional capacity of TMS can be used to distinguish between competing accounts about how specific cognitive processes arise neurally. Specifically, the question is: To what extent can these approaches provide novel insight about cognition beyond established uses of TMS for structure–function relationships? Can TMS also be used to test for the requirement of specific regional interactions in cognition, and can TMS-evoked changes in such interactions allow for disambiguating competing cognitive theories?

One such example has already been provided, in which concurrent TMS–fMRI was used to adjudicate between two competing mechanisms (of prefrontal-based control) in working memory.^113^ The physiological predictions (in terms of BOLD signal increase in memory target areas) matched the expected outcome corresponding to one of two theoretical functions for top-down control (enhancement versus suppression). With the causal conclusions permitted by TMS, this study provides a strong line of evidence favoring a specific (theoretically described) implementation of cognitive control. Furthermore, other combined offline TMS–fMRI^12^ and TMS–EEG studies^120^ revealed other prefrontal, but anatomically distinct, regions also involved in the control of task relevant versus irrelevant information. This raises the question for future studies about the specific roles of these three prefrontal regions, and whether they indeed fulfill essentially the same function. Alternatively, enhancement and suppression could be differentially invoked under different conditions, by different brain regions (e.g., when items are already in WM but face exogenous distractors versus when items among distractors are being encoded, or need to be selected from among already encoded items that have become distractors).

Note that an experiment utilizing concurrent TMS–fMRI does not automatically confer an informative contribution to a (neuro)cognitive model. The danger of simply applying an anatomical label to a function, albeit a causal one, remains (although, to some degree, this problem applies to all TMS approaches). However, concurrent TMS-neuroimaging does hold a unique position through its ability to produce specific context-based modulations of physiological signals with direct and immediate effects able to be observed. Another strength of concurrent TMS–neuroimaging is that it provides strong evidence about network accounts of cognition (i.e., how cognitive behaviors arise through concerted communication between brain regions, some of which may enact control over others). As addressed below, the possible directionality of signals can be explored with formal models of effective connectivity. However, we caution against the fallacies of reverse inference. It will not always be possible to unambiguously link TMS-induced changes in neural activity and cognitive processing, particularly in cases where behavioral perturbations do not occur. While it may be safe to conclude that the TMS-induced impact on region A and its interconnected regions B and C varies as a function of cognitive state (e.g., attention), one cannot conclude that attention is enabled because of the interactions between A, B, and C.

### Combined TMS–neuroimaging and multivoxel pattern analyses

Multivoxel pattern analysis (MVPA) is a recently developed approach to the analysis of neuroimaging data that shows the information content of signals in high dimensional data sets, such as those of fMRI, EEG, and magnetoencephalography (MEG). Using machine learning, MVPA can decode the information contained in a signal, revealing the nature of how a brain region or an electrophysiological signal represents that information, and how it can change over different stages of processing.^136^ For example, returning to the experimental question of whether the DLPFC enhances memory targets or suppresses distractors during working memory,^113^ disrupting the DLPFC might result in the representation of targets being affected such that the (MVPA-identified) representations are decreased as a result of the disturbance of top-down enhancement. Similarly, if distractor suppression is also a DLPFC-based control mechanism, disruption of this might result in the improvement of distractor decoding, due to the lifting of suppression (unpublished data, Feredoes *et al*.). Given that the technological requirements for combining TMS with MVPA are already in place, the experimenter is limited merely by their ability to formulate questions appropriate for this approach; the ability to observe TMS-induced modulation of information representation may significantly increase the inferential power afforded by combined TMS–neuroimaging.

### Computational neurostimulation

Given the causal inferences afforded by TMS, it may be integrated with emerging classes of connectivity models, as a way to test their parameters and/or validity as a whole. Recent appraisals of neuroimaging highlight the shift from establishing functional specialization of regions toward establishing the connections between regions instead, and thus functional integration;^137^,^138^ with this has come the desire to establish causal links between such connected regions via neurobiologically informed models that provide intermediate levels of description about the basic physiology of TMS and its behavioral consequences.

For example, biophysical models, such as those instantiated in dynamic causal models (DCMs), can in principle help to test whether remote stimulation effects predominantly arise from ortho- and/or antidromic stimulation of connections, and/or via pyramidal projections or intra-regional interneurons.^53^,^55^,^139^–^141^ In principle, DCMs for electrophysiological data, such as provided by MEG or EEG, are best equipped to address such mechanistic questions, for example, by forming deterministic generative models of ensemble or population dynamics.^142^ DCMs now exist for evoked responses,^143^ steady state responses,^144^ cross-spectral densities,^145^ phase coupling,^146^ and induced responses,^147^ thus offering complementary ways for investigating how TMS elicits observed population responses, but also how such responses affect cognitive processing.

One can furthermore proceed to ask about competing candidate mechanisms by which cognitive states might be maximally expressed in effective connectivity changes, and how these changes then interact with neurostimulation. For example, DCMs also test for TMS-induced changes in physiological responses, and their interaction with cognition. By estimating parameters of the neural model, one might expect that the most accurately predicted physiological signals would correspond closely to the observed fMRI/EEG/MEG signals. Put simply, a realistic biophysical model should also be able to explain an additional input into the system in the form of a TMS-induced perturbation, and in this way, a causal intervention dimension can be added to modelling. In other words, the combination of *in vivo* perturbation through neurostimulation, neuroimaging, and realistic brain models provides a computational neurostimulation approach with which to investigate the impact on cognition of physiological mechanisms of neurostimulation-induced effects.^148^,^149^

In what could be considered a precursor to such an approach, one study^150^ investigated how TMS-evoked interregional influences within motor and corticolimbic circuits change with drug-induced global state changes. Using DCMs to investigate TMS-evoked changes in effective connectivity and their interaction with drug treatment, drug-specific changes were demonstrated in the brain networks targeted by TMS. This provides an example of how concurrent TMS and neuroimaging can quantify drug-induced changes in interregional interactions (Fig. 4A). More significantly, it illustrates how concurrent neurostimulation and neuroimaging can be used together with neurobiologically informed analyses of effective connectivity to ask questions about the rapid and flexible causal network interactions during cognition.

### TMS and entrainment

The previous sections described how TMS can entrain α oscillations and how this entrainment relates to endogenous attention^134^ (Fig. 4B). This application of TMS promises a direct and therefore powerful way in which to investigate how oscillations might be integral to cognition. The seemingly fortuitous ability of TMS to be applied rhythmically to match natural oscillations of various frequencies means a variety of questions are ready to be answered using this approach. For example, the proposed long-range communication role for the lower frequencies^151^ can be tested by applying TMS over one region and measuring the resultant oscillatory changes in distant, connected regions. Moreover, improvements in behaviors can also be produced, via the boosting of frequency-specific amplitudes with TMS.^152^ However, for this latter point, transcranial alternating current stimulation techniques may be an alternative and potentially more powerful way for inducing behaviorally relevant entrainment,^153^–^158^ mainly because of their potential to entrain at high (e.g., γ range) frequencies.^159^ Recent developments in high-definition tDCS for increased focality of stimulation may also enable more selective targeting of cortical regions.^160^–^164^ Moreover, recent advances in forward modelling of induced currents now allow, in principle, precise directing of induced currents onto the desired neural target structures.^162^,^165^ Together, these developments hold promise for the use of tDCS in locally constrained neural entrainment.

Thus, we predict a rapid growth in studies using neurostimulation to understand the role of specific frequencies of oscillations for specific cognitive processes and for entrainment to facilitate their function.

### Neurostimulation and neurotransmitters

**Figure 5:**
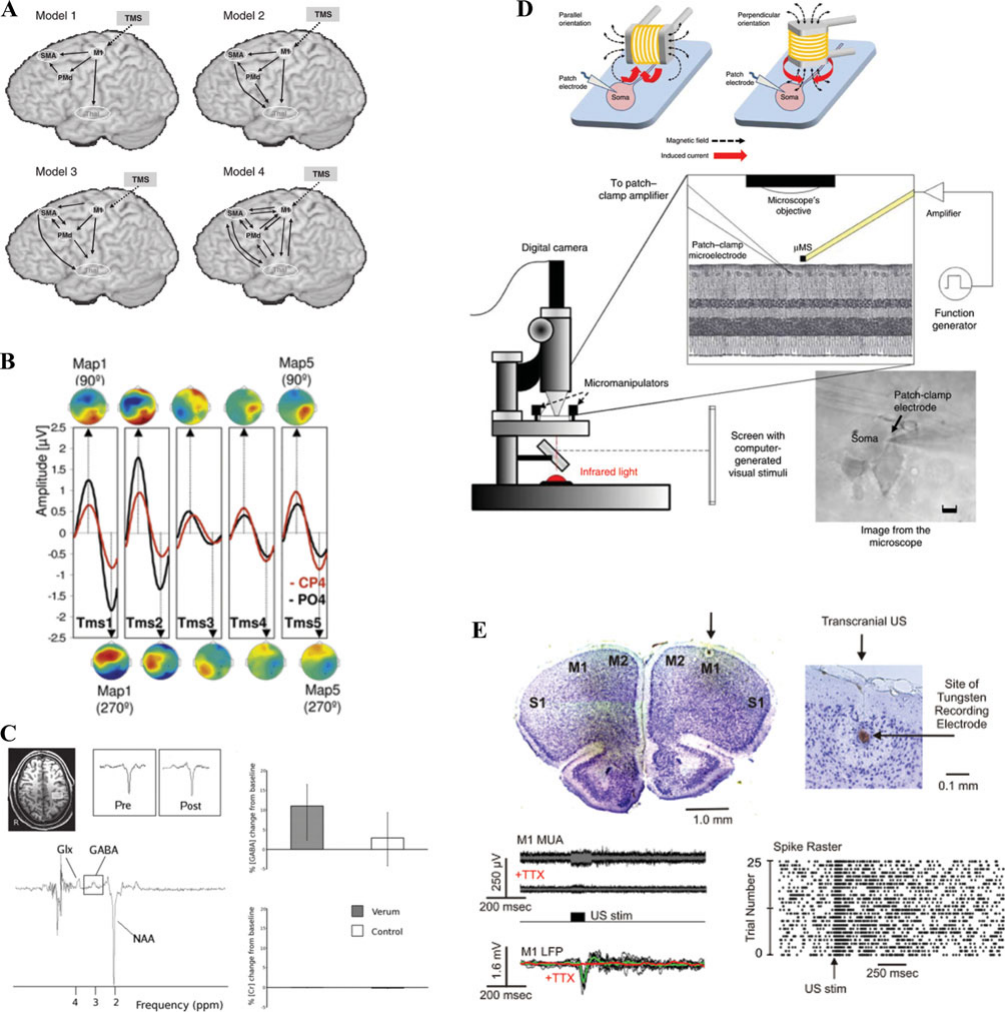
The future of concurrent neurostimulation and neuroimaging in humans for studies of cognition. (A) Network analyses such as dynamic causal modeling provide intermediate levels of description that link the physiological impact of neurostimulation and its behavioral consequences, and allow for generating testable hypotheses about resulting network connectivity changes. Adapted from Li *et al*. 150 with permission. (B) Neurostimulation-induced entrainment of intrinsic cortical rhythms can have direct consequences for perception, depending on the frequency of stimulation. This allows for identifying the causal role of oscillatory activity for behavior, and holds the possibility to shape behavior through the selective entrainment of local and distributed oscillatory activity. Adapted from Thut *et al*.^134^ with permission. (C) Combined magnetic resonance spectroscopy can quantify the specific neurotransmitter changes induced by neurostimulation, potentially including online neurostimulation protocols. Moreover, the changes can be directly related to behavior, and thereby provide causal links between stimulation-evoked neurotransmitter changes in focal and defined parts of the brain, and cognition. Adapted from Stagg *et al*.^167^ with permission. (D) Micro-stimulation coils in principle allow for selective stimulation of cortical micro-circuits, and include the possibility for application during neuroimaging Adapted from Bonmassar *et al*.^181^ with permission. (E) Ultrasound stimulation holds promise to allow for targeted and selective stimulation of neural tissue throughout the brain, including subcortical structures. Adapted from Tufail *et al*.^184^ with permission. Note that for the examples in D–E, demonstration of their applicability in humans is pending.

The burgeoning development of novel imaging and neurostimulation approaches is likely to continue to fuel studies that address hitherto inaccessible questions. For example, the abovementioned DCMs empower one to use anatomically constrained computational perturbations of specific neurotransmitters (i.e., testing hypotheses regarding neurotransmitter changes throughout the brain evoked by neurostimulation). Importantly, such changes can also be quantified both directly at the stimulation site and throughout the brain with magnetic resonance spectroscopy (MRS).^166^ MRS allows quantification of neurotransmitter concentrations within a defined region of interest in the brain and has recently been used to measure both TMS- and tDCS-induced neurotransmitter changes (albeit in offline approaches)^167^–^171^ (Fig. 5C). Moreover, it has become clear that interindividual variation in, for example, MRS-measured GABA levels relate to variation in task performance in a number of regions.^172^–^176^ This now opens up possibilities to address questions about causal neuropharmacology by changing neurotransmitter concentrations in a defined cortical network through neurostimulation, and relating such changes to task performance. One crucial advantage of this approach is that, in principle, neurotransmitter concentrations can be modified in a more spatially selective way than otherwise possible with pharmacological interventions, although it remains to be seen whether MRS can be usefully combined with concurrent neurostimulation. Currently, the technology requires a priori focus on specific neurotransmitters (e.g., GABA and glutamate/glutamine (Glx)), despite the known impact of both TMS and tDCS on a larger variety of neurotransmitters,^2^,^177^–^179^ and additionally provides a relatively poor spatial resolution (compared to fMRI and the focality of TMS), with standard voxel-sizes at field strengths of 3T of around 30 mm^3^. This inevitable sampling-bias currently constrains inferences about causal neurotransmitter–function relationships, but advances in multivoxel MRS^180^ (particularly at higher field strengths) are likely to provide increasingly detailed mappings of stimulation-evoked changes in neurotransmitter concentration with relevance for cognition.

### Novel ways for neurostimulation

Other, potentially more powerful and more focal neurostimulation techniques may be in the offing and invite more speculative projections. Recent work demonstrates the ability of micro-magnetic stimulation (μMS) coils with dimensions of around 500 μm to stimulate retinal ganglion cells^181^ (Fig. 4D). Such coils can be used *in vivo* and *in vitro* with high focality, and are inherently compatible with neuroimaging. Although it remains to be shown that they can be usefully applied in human studies, the possibility of selective stimulation of small neural circuits appears achievable. Other developments hint at the possibility of low-intensity–focused ultrasound pulsation to interact with neural processing,^182^,^183^ at a spatial scale of 2 mm or less^184^ (Fig. 4E). Pending demonstration that this technique can be safely applied in humans to stimulate brain tissue, focal stimulation beyond the range of present technology during neuroimaging may become a possibility, again being potentially compatible with neuroimaging, including MR-based techniques.^182^ The increasing feasibility of optogenetic approaches for studies of behavior^185^ may, in the future, provide yet another powerful avenue for neurostimulation. Optogenetic stimulation is the excitation or inhibition of specific cell types and neural pathways through light pulses. This provides a causal, time-resolved assay in which to test for the contribution of specific neural circuit elements that participate or contribute to the computations required for emergent behavior; optogentically controlled stimulation has recently been used in conjunction with fMRI to investigate the foundations of the BOLD fMRI signal.^186^ Any transfer of this technique to humans is likely to be at least a decade away, though recent work suggests that translational applications could become feasible within such a time frame,^187^ and, if that is the case, such applications could provide auxiliary insights into the neural underpinnings of human cognition. It is not clear whether and/or when some of these techniques will be safely transferrable to humans, but some of the rapid developments will, in some form, make the transition to human studies of cognition and the neural networks that underpin behavior. This review does not cover an exhaustive list of possibilities in which neurostimulation and neuroimaging might be applied in the future, but illustrates how extant combinations of these methods already offer us an expanded set of tools and a rich vein of information with which to interrogate and reconceptualize how complex neural network interactions can give rise to behavior.

## Conclusions

This review has illustrated the increasing sophistication of studies combining neurostimulation with neuroimaging, as well as an exciting future outlook, for studies of human cognition. As cognitive neuroscience integrates with more fundamental aspects of neurophysiology, we suggest that neurostimulation will have an important role to play, given that it modulates neurophysiology at a fundamental level. The shift in the use of neurostimulation to study cognition is already apparent, via the more complex combinations and experimental questions and designs using combined neurostimulation-neuroimaging, some of which we have described above. By continuing in this direction, along with developments in the field of neurostimulation, the current possibilities and future developments hold promise to establish a causal neurocognition account in the human brain.

## Footnotes

a Perhaps we owe the strength of such distal effects to the fact that TMS interacts with the activity of relatively large populations of neurons, compared to combined neuroimaging and microstimulation experiments in nonhuman primates.^188^–^194^ The exact extent of the functional impact depends on many parameters, but can be as little as 0.5–1 cm^2^,^195^ particularly when combined with neuronavigation.^196^,^197^ But, as opposed to microstimulation in nonhuman primates, the stimulation of much larger populations of neurons may in fact be a prerequisite for substantial distal effects elicited by TMS.
